# Inferring MicroRNA Activities by Combining Gene Expression with MicroRNA Target Prediction

**DOI:** 10.1371/journal.pone.0001989

**Published:** 2008-04-23

**Authors:** Chao Cheng, Lei M. Li

**Affiliations:** 1 Molecular and Computational Biology Program, Department of Biological Sciences, University of Southern California, Los Angeles, California, United States of America; 2 Department of Mathematics, University of Southern California, Los Angeles, California, United States of America; Children's Hospital Boston, United States of America

## Abstract

**Background:**

MicroRNAs (miRNAs) play crucial roles in a variety of biological processes via regulating expression of their target genes at the mRNA level. A number of computational approaches regarding miRNAs have been proposed, but most of them focus on miRNA gene finding or target predictions. Little computational work has been done to investigate the effective regulation of miRNAs.

**Methodology/Principal Findings:**

We propose a method to infer the effective regulatory activities of miRNAs by integrating microarray expression data with miRNA target predictions. The method is based on the idea that regulatory activity changes of miRNAs could be reflected by the expression changes of their target transcripts measured by microarray. To validate this method, we apply it to the microarray data sets that measure gene expression changes in cell lines after transfection or inhibition of several specific miRNAs. The results indicate that our method can detect activity enhancement of the transfected miRNAs as well as activity reduction of the inhibited miRNAs with high sensitivity and specificity. Furthermore, we show that our inference is robust with respect to false positives of target prediction.

**Conclusions/Significance:**

A huge amount of gene expression data sets are available in the literature, but miRNA regulation underlying these data sets is largely unknown. The method is easy to be implemented and can be used to investigate the miRNA effective regulation underlying the expression change profiles obtained from microarray experiments.

## Introduction

MicroRNAs (miRNAs) are small non-coding RNAs of 19–24 nucleotides in length that down-regulate gene expression during a variety of crucial cell processes, including cell proliferation [1], apoptosis [2], development [3], differentiation [4], and metabolism [5]. Since the discovery of the first miRNA gene (lin-4) in 1993 [6], several hundreds of miRNA genes have been identified in animals and plants. It is currently estimated that miRNA genes constitute about 1%–2% of the known genes and up to 30% of genes may be regulated by miRNAs in eukaryotes [7]. MiRNA genes can be located in introns or exons of protein-coding genes, or within the intergenic regions between protein-coding genes. They can either exist individually or form polycistronic clusters [8]–[10]. Mature miRNAs are originated from 70- to 100-nucleotide hairpin pre-miRNA precursors [9]. Typically, a precursor is cleaved by RNase III family of endonucleases Drosha and Dicer into a duplex: one strand of the duplex is degraded, whereas the other strand functions as mature miRNA [11]. The single-stranded mature miRNA is then incorporated into a silencing complex and guided to the 3′ un-translated region (3′-UTR) of the target mRNAs via base pairing, leading to the block of translation or degradation of the target mRNAs [12].

It has been thought that the degree of complementarity between the miRNA and its target mRNA determines the fate of the bound target mRNA [13]–[15]. Perfect pairing induces target mRNA cleavage, as is the case in most plant miRNAs [16], [17]. Imperfect pairing in the central part of the duplex instead leads to the block of translation, as seen in the majority of animal miRNAs [18], [19]. More recent studies, however, have demonstrated that in both plants and animals, expression regulation at the mRNA level (via mRNA degradation or deadenylation) may serve as a common mechanism for miRNA function. First, animal miRNAs have been found to mediate mRNA degradation even when the target sites have incomplete complementarity to them [20]–[22]. Second, microarray experiments reveal that overexpression of miRNA in cells cause the moderate down-regulation of a large number of transcripts, many of which contain the complementary sequences of the over-expressed miRNA in their 3′-UTRs [23]–[25]. Conversely, gene expression analysis from miRNA knockdown animals reveal that the miRNA recognition motifs are strongly enriched in the 3′-UTRs of up-regulated genes, but depleted in the 3′-UTRs of down-regulated genes [26]. Third, it has been shown that the 3′-UTRs of certain class of ubiquitously expressed genes are specifically depleted of miRNA target sites [27] and that the endogenous expression of several highly specific miRNAs is typically negatively correlated with the mRNA levels of their targets [27]–[29].

Since the expression regulation at the mRNA level is common for miRNA functions, it is reasonable to expect that the activities of miRNAs can be reflected by the expression levels of their target mRNAs. As a matter of fact, the systematic negative correlations between expressions of miRNAs and those of their target transcripts have been observed in a number of studies as described above [23]–[29]. With these observations, a natural question to us is: can we infer the modification of miRNA effective regulation from the expressions of their target genes? In this paper, we propose a method that combines microarray expression data with miRNA target predictions to infer the relative activities of miRNAs underlying the gene expression changes. In a typical microarray expression experiment, the relative expression levels in two different biological samples (cDNA arrays) or the absolute expression levels in a single biological sample (oligonucleotide arrays) are measured simultaneously for tens of thousands of genes. The expression levels of miRNA are generally not available from these gene expression data. Thus, the purpose of our method is to infer the relative miRNA activities, i.e. changes of miRNA effective regulations between two different conditions, based on the expression changes of their target genes, which are directly measured by cDNA arrays or calculated by comparing the absolute gene expression levels from different oligonucleotide arrays. Basically, our method examines the trend of expression changes of target genes of a miRNA. If these target genes tend to be down-regulated, it indicates that the effective activity of this miRNA is enhanced between the two conditions. Conversely, a prevalent up-regulation of these target genes would indicate a reduction of the miRNA activity. We apply this method to microarray data measuring gene expression changes in cell lines transfected with certain miRNAs or anti-miRNAs (miRNA-specific inhibitors). It shows that the relative activities changes of the transfected miRNAs and the inhibited miRNAs can indeed be inferred with high sensitivity and specificity. The method is easy to be implemented and can be applied to other microarray data to provide useful information regarding the underlying miRNA activity regulations.

## Results

We apply our method to the microarray expression data from miRNA transfection experiments performed by Lim et al. [23]. In this data, using non-transfected Hela cell as reference the relative expression levels of genes are measured in the HeLa cells at the 12 (12 h) and 24 (24 h) hour after respective transfection with two wild-type miRNAs (miR-1 and miR-124), two mutant miRNAs (124mut5-6 and 124mut9-10) and two chimeric miRNA (chimiR-124/1 and chimiR-1/124), which results in a total of 12 expression change profiles. We applied our method to these 12 expression change profiles to examine whether the activity enhancement of the transfected miRNAs can be detected. By integrating the expression data with the miRNA target prediction data, we infer the activity change scores (AC scores) with statistical significances for 211 human miRNAs in each of the 12 expression change profiles. More detailed description about the data, the definition of AC score, and the computational procedure can be found in section “Materials and Methods”. The AC score is a measure of the inferred relative activities of miRNAs between two different conditions. In other words, it estimates the capability of these miRNAs to down-regulate expressions of their target genes. A positive AC score indicates activity enhancement of the corresponding miRNA, whereas a negative AC score indicates activity reduction. The complete results for 211 human miRNAs in these 12 expression change profiles are shown in the supplementary Table S1.

### Relative activities of miRNAs in miRNA transfected HeLa cells

**Figure 1:**
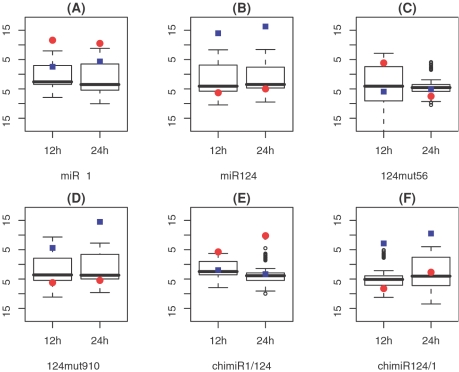
Distribution of AC scores for 211 known human miRNAs in (A) miR-1 (B) miR-124 (C) 124mut5-6 (D) 124mut9-10 (E) chimiR1/124 and (F) chimiR124/1 transfection data. The AC scores for miR-1 and miR-124 are marked as red circles and blue rectangles, respectively.

In Figure 1, we show the distribution of the AC scores for these 211 human miRNAs using box-plots, in which miR-1 and miR-124 are marked as red circles and blue rectangles, respectively. As shown, the regulation of miR-1 (Figure 1A) and miR-124 (Figure 1B) in their transfection microarray profiles are both detected by our method. In the miR-1 transfection data, the AC score of miR-1 is 12.37 at 12 h and 10.37 at 24 h. In the miR-124 transfection data, the AC score of miR-124 is 14.16 at 12 h and 16.11 at 24 h. These four AC scores are the highest among the 211 scores inferred from the corresponding miRNA transfection experiment, with q-values of 0 according to the results from 10,000 permutations.

Figure 1C indicates that switch of bases at position 5 and 6 (124mut5-6) substantially reduces the ability of miR-124 to down-regulate its target genes. In the 124mut5-6 transfection experiment, the AC score of miR-124 is −5.89 at 12 h (q = 0.0084) and −5.22 at 24 h (q = 0.10), suggesting slight reduction instead of an enhancement in regulatory activity. It seems that the regulatory activity of miR-124 is completely abolished as a consequence of base switch between positions 5 and 6. In contrast, switching bases between positions 9 and 10 (124mut9-10) exhibits less reduction of regulatory activity. As shown in Figure 1D, at 24 h the AC score for miR-124 in 124mut9-10 transfected HeLa cells is 14.15 (q = 0), which is comparable to 16.11 (q = 0) in the wild-type miR-124 transfected HeLa cells. However, the AC score of miR-124 at 12 h after the 124mut9-10 transfection is much lower (5.28, q = 0.063). This indicates that expression of the target genes of miR-124 is only partially down-regulated at 12 h due to the lower regulatory activity of 124mut9-10. Therefore, our results demonstrate that the silencing or down-regulation abilities of both 124mut5-6 and 124mut9-10 are reduced compared to miR-124. Furthermore, positions 5–6 of the miRNA are more crucial than positions 9–10.

Figure 1E and 1F show that the 5′-ends of the miRNAs are dominant over the 3′-ends in down-regulating target genes. As shown, transfection of the chimaeric miRNA chimiR-1/124, composed of 5′-end of miR-1 and 3′-end of mir-124, down-regulates the expression of the target genes of miR-1; whereas transfection of the chimaeric miRNA chimiR-124/1, composed of 5′-end of miR-124 and 3′-end of mir-1, down-regulates the expression of the target genes of miR-124. These results are consistent with previous studies which demonstrated that the miRNA-target RNA interaction is restricted to the 5′-end of the miRNA sequence and that the perfect complementarity between the miRNA 2–8 bases and the targeted RNA is essential for target recognition [30]–[33].

**Figure 2:**
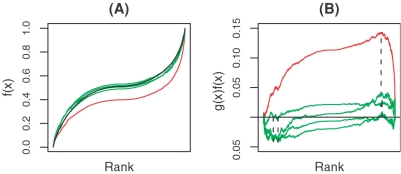
Examples of pre-score calculation for miR-124 and four randomly selected miRNAs in the miR-124 transfection profile at 24 h. The *g(i)* function is shown as the black curve in (A); the *f(i)* functions in (A) and the *g(i)–f(i)* function in (B) for miR-124 and the randomly selected miRNAs are shown as the red lines and green lines, respectively. The dashed lines show the positions at which maximum deviations (pre-scores) are achieved for these miRNAs.

In Figure 2, we use the expression change profile at 24 h after miR-124 transfection as an example to illustrate how our method works. Figure 2A shows the *g(i)* function in Equation (1) (black curve) and *f(i)* functions in Equation (2) for miR-124 (red curve) and four randomly selected miRNAs (green curves). The definitions of *g(i)* and *f(i)* functions can be found in “Materials and Methods”. If the activity of a miRNA does not change, *f(i)* would increase in a random fashion. However, if the activity of a miRNA does change, say in a down-regulated way, *f(i)* would increase rapidly. The function *g(i)* serves as a control for the comparison with *f(i)*. As shown in Figure 2A, the *f(i)* function for miR-124 exhibits a curve which deviates far from the curve of *g(i)* function, since the genes with high binding affinities to the miRNA tend to be down-regulated and therefore are enriched at the bottom of the sorted expression change profile *e*′. However, the *f(i)* functions of these randomly selected miRNAs are indistinguishable from the *g*(*i*) function. Thus, the maximum deviation of *f(i)* from *g(i)*, i.e. the pre-score, for miR-124 is much larger than those of the four randomly selected miRNAs, as shown in Figure 2B.

Interestingly, we find that over-expression of miR-1 or miR-124 can cause the activity changes of some other miRNAs, which are mostly down-regulated. For example, after miR-124 transfection, the activity of miR-32 is down-regulated with an AC score of −10.05 (q = 0) at 12 h and −9.57 (q = 0) at 24 h. These results support the notion that miRNAs regulate each other and form a regulatory network like transcription factors [34]. An alternative explanation, however, is that the large amount of exogenous miRNA introduced by miR-1 or miR-124 transfection may interfere with the processing and maturation of endogenous miRNAs and lead to their down-regulation.

We also applied our method to the shuffled expression change profiles, in which the gene names in the microarray data are shuffled, or in other word, mis-labeled randomly. In the shuffled data, we are unable to identify any miRNA with significant activity change at 0.30 significance level (q<0.30), suggesting a high specificity of our method.

### Robustness with respect to false miRNA target predictions

**Figure 3:**
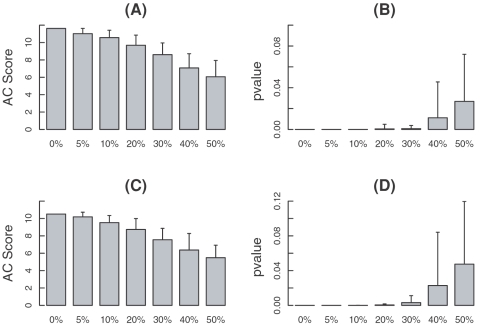
Average AC scores and p-values for miR-1 in the miR-1 transfection profile at 12 h (A and B) and 24 h (C and D) based on perturbed miR-1 target prediction scores. The x-axis shows the perturbing percentage from 0% to 50%. At each perturbing percentage, 100 perturbed binding score profiles of miR-1 are produced by exchanging the binding scores of target and non-target genes of miR-1. The standard deviations of AC scores and p-values are also shown.

It is known that *in silico* miRNA target prediction is usually not accurate. Depending on the cut-off setting, the false positive rate and/or the false negative rate of the target predictions could be fairly high. Nonetheless, our method achieves accurate inference of miRNA activity modification in the miRNA transfection data as shown above. To investigate the robustness of our method to the false miRNA target predictions, we introduce additional errors to the miRNA target prediction data and examine whether our method is still able to identify the activity enhancement of the transfected miRNAs. By setting the cut-off value of binding energy to −12, the miRanda algorithm predicted 1076 regulatory target genes for miR-1 (transcripts corresponding to the same gene are combined). We divide the genes into a target gene set and a non-target gene set of miR-1. To introduce additional prediction errors, we randomly select 5%, 10%, 20%, 30%, 40% and 50% genes from the target gene set, set their miR-1 binding scores to 0s and assign their original binding scores to an equal number of randomly selected non-target genes. In other words, we swap the binding sores of a certain percentage of genes in miR-1 target and non-target gene sets. We then calculate the AC score of miR-1 in the expression change profile at 12 h and 24 h after miR-1 transfection based on the perturbed binding affinity data. For each percentage, we repeat the above procedure 100 times. The resulting average AC scores of miR-1 at each perturbing percentage and their p-values are shown in Figure 3.

As shown in Figure 3, the average AC scores decrease gradually with the increase of perturbing percentage. The activity change of miR-1, however, can still be detected even when the perturbing percentage increases to as large as 30%. Considering that the original miRNA binding data already contain some prediction errors, we conclude that our method is robust to the false positive predictions in the predicted miRNA binding affinity data.

### Continuous versus discretized miRNA-target binding score data

**Figure 4:**
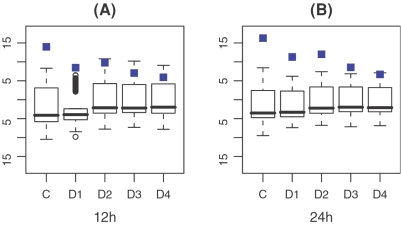
Box-plots of AC scores for the 211 miRNAs in the miR-124 transfection profiles at 12 h (A) and 24 h (B) based on the continuous and discretized binding score data. C corresponds to the continuous binding score data; D1, D2, D3, and D4 correspond to the discretized binding score data with cut-off value of 12, 60, 80 and 100, respectively. The AC score for miR-124 is marked as blue rectangle.

In our method, we directly utilize the continuous binding scores of miRNAs to their targets. To identify miRNAs with significant activity changes, other strategies can be used: define the target gene set for each miRNA and then (1) perform gene set enrichment analysis (GSEA) [35] or (2) use the Wilcoxon test to compare the expressions of target genes with non-target genes. To show the advantage of using continuous binding scores, we discretize the binding affinity data by setting the binding scores over a cut-off value to 1 and those below the cut-off value to 0. When discretized binding scores are used, our method is essentially similar to the GSEA method. We apply our method to the discretized binding affinity data and the expression change profiles. Figure 4 shows the results based on the discretized binding score data with different cut-off values. As shown, the results based on continuous binding scores outperform those based on discretized data.

We also use the Wilcoxon test to examine the significance of down-regulation of target genes (binding score ≥12) for each miRNA. It turns out that the down-regulation of the target genes of the transfected miRNA can be detected, but the specificity is relatively low. For example, in the expression change profile at 12 h after miR-1 transfection, the p-value of miR-1 by the Wilcoxon test is 0.0021, indicating the down-regulation of miR-1 target genes. However, there are 20 other miRNAs with more significant p-values, the lowest being 4.2e-11. In the expression change profile at 24 h after miR-1 transfection, the p-value of miR-1 is 0.061 and as many as 46 miRNAs have more significant p-values with the lowest at 7.2e-11. Therefore, our method, which is based on the continuous miRNA-target binding score data, achieves more accurate results than those methods based on miRNA target gene set analysis.

### Other MiRNA transfection/inhibition data sets

**Figure 5:**
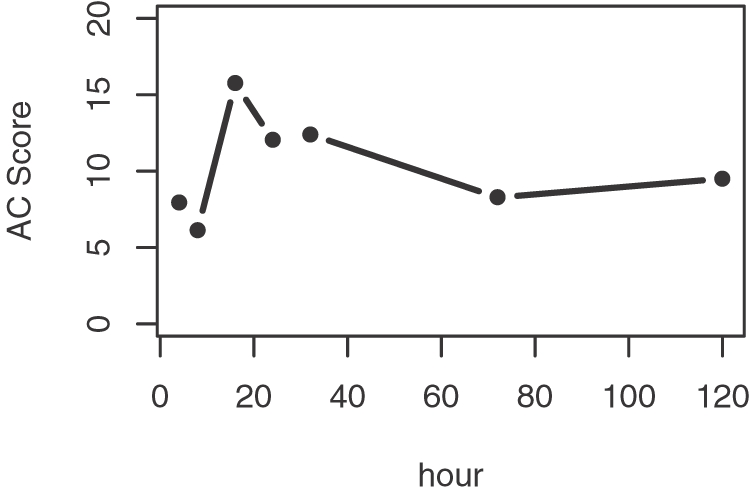
The inferred AC scores of miR-124 in the microarray time course of miR-124 transfection.

In addition to the data described above, we apply our method to two other microarray data sets from miRNA transfection or inhibition experiments. The first data set is from the miRNA transfection experiment [24]. This data set is different from the previous one in two aspects. First, it takes a time course design, and measures gene expressions at 7 time points from 4 h to 120 h after miRNA transfection. Second, it uses one-channel Affymetrix microarrays to measure absolute expression levels of genes. In this data set, two time-course microarray experiments are included: one for miR-124 transfection and the other for the negative control transfection. Comparison of gene expressions in these two time courses at all time points results in 7 expression change profiles, which reflect expression changes caused by miR-124 transfection at different time points. We calculate the AC scores and their significances for each of the 211 miRNAs in these profiles. In Figure 5, we show the inferred relative activities of miR-124 across the time course after its transfection. As shown, moderate activity enhancement of miR-124 is observed at 4 h and 8 h with AC scores of 7.96 (q = 9.4E-4) and 6.15 (q = 0.026), respectively. In the rest time points (up to 120 h after transfection), we detect significant enhancement of miR-124 activity, with the highest AC score of 15.75 (q = 0) achieved at 12 h. The complete results for this data set can be found in the supplementary Table S2.

In the second data set, the effects of over-expression and inhibition of two human miRNAs, miR-16 and miR-106b, are investigated using the two-channel Agilent microarrays [25]. We apply our method to this data set and the complete results can be found in the supplementary Table S3. We detect the significant activity enhancement of miR-16 and miR-106b in Hela cells transfected with the corresponding miRNA, with the AC scores for miR-16 and miR-106b are 19.08 (q = 0) and 21.31 (q = 0), respectively. Of more importance, in the HeLa cells where the endogenous miR-16 and miR-106b levels are specifically inhibited by 2′-O-methyl-modified oligonucleotides (anti-miR), we detect the activity reduction of these two inhibited miRNAs. The AC scores of miR-16 and miR-106b are respectively −16.00 (q = 0) and −10.30 (q = 0) in the miRNA inhibition experiments. As suggested by these results, our method is able to detect the activity change of miRNAs in both gain-of-function (miRNA transfection) and loss-of function (miRNA inhibition) experiments.

## Discussion

In this study, we propose a computational method to infer miRNA effective activity by integrating their binding affinities to target genes with gene expression changes measured by microarray experiment. With this method, we successfully detect activity enhancement of the miRNAs transfected to HeLa cells with high sensitivity and specificity. It should be noted that expression changes of the target genes for a miRNA reflects its effective regulatory activity change rather than expression change. This is one of the advantages of our method, since the expression level of a miRNA may not reflect its ability to down-regulate target genes. First, miRNA precursors need to be processed to become mature miRNA and post-transcriptional regulation may play important roles in controlling miRNA activities [36]. Second, in some cases such as cancer, mutation of miRNA sequences may cause activity changes without substantially changing the expression levels. For these reasons, it may be more useful to measure or infer miRNA activity changes instead of measuring their expression changes. Unfortunately, some miRNA quantification methods such as miRNA microarray cannot effectively discriminate the expression levels of pre-miRNAs from those of mature miRNAs [37], [38]. Thus the miRNA expression levels measured by these methods do not reflect the actual regulatory activities of mature miRNAs. Furthermore, the number of large-scale miRNA profiling data is still limited, whereas a huge amount of microarray gene expression data are available from the public database such as Stanford Microarray Database (SMD) [39] and Gene Expression Omnibus (GEO) [40]. Our method may be implemented to these data sets and provide insight into miRNA activity regulation for further experimental investigation.

In this study, the target genes for miRNAs were predicted using miRanda algorithm [41]. The self-consistency in our analysis of the miRNA transfected HeLa cells suggests the reliability of the miRanda predictions. Other than the miRanda algorithm, several other target prediction methods have also been proposed such as TargetScan [30], DIANA-MicroT [42], and PicTar [43]. We do not study the effect of different miRNA target prediction methods, since it is beyond the focus of this paper. As shown, our method is robust with respect to the possible false predictions in the miRNA-target binding score data. However, we expect further improvement of the results by our method if more accurate miRNA target gene prediction is available.

Our method relies on the connection between activity of a given miRNA and expression levels of its target genes. Although recent studies indicate that expression regulation at the mRNA level may be a common mechanism for miRNA function in both plants and animals, the debate about the regulatory mechanisms of animal miRNAs is far from being closed. It is possible that for some animal miRNAs translation repression is the major mechanism for target gene suppression. In this case, our method may not be able to detect the activity changes of these miRNAs based on gene expression data. Another limitation of our method is that different miRNAs are considered independently during the activity inference. But miRNA regulation may involve interactive activity between multiple miRNAs. How to take into account the synergistic miRNA regulation would be an interesting direction in the future studies.

In summary, we propose a method to infer effective activities of miRNA from microarray gene expression data and miRNA target predictions. This method can detect the activity enhancement of transfected miRNAs as well as the activity reduction of inhibited miRNAs with high sensitivity and specificity based on gene expression data. Since a huge amount of microarray expression data is available, we expect this method can be applied to infer miRNA regulations in many biological and medical research.

## Materials and Methods

### Overview

We originally use a similar method to infer activity changes of transcription factors by integrating microarray data with ChIP-chip data or motif discovery data [44]. Since transcription factor and miRNA share a common logic in gene expression regulation [34], in this paper we revise the method and apply it to infer the relative activities of miRNAs by combining gene expression profiling with miRNA target predictions. In a typical expression change profile, the expression changes of tens of thousands transcripts are measured simultaneously under two different conditions, i.e. mutant versus wild type. To infer the relative activity of a miRNA, we examine expression changes of its target transcripts: if they tend to be down-regulated, then the activity of this miRNA is likely to be enhanced; conversely, if they tend to be up-regulated, then the activity of this miRNA is likely to be repressed. Unfortunately, the complete list of target transcripts of miRNAs is usually not available from experiments. But a number of computational approaches have been suggested to predict miRNA targets, such as TargetScan [30], miRanda [41], DIANA-microT [42], RNAhybrid [45], and PicTar [43]. These approaches search the 3′-UTR sequences of mRNAs for potential miRNA binding motifs that have binding affinities to the miRNA over a cut-off value. In our method, we do not use a stringent cut-off value to determine target gene sets for miRNAs. In contrast, we utilize the predicted binding affinity scores directly, for the magnitude of binding affinities itself is informative as has been shown by [24].

### Human miRNA target prediction

In plants, miRNAs are highly complementary to their binding sequences and therefore miRNA target prediction is relatively easy [46]. In animals, a number of miRNA target prediction approaches have been suggested in the past few years [30], [41]–[43], [45], [47]. In this paper, we utilize the data predicted by miRanda algorithm [41], [47], which include the target predictions for 211 of known miRNAs in human. The complete data set is available from the miRNAMap database at http://mirnamap.mbc.nctu.edu.tw/html/downloads.html [48]. The miRanda algorithm is based on sequence complementarity between the mature miRNA and its target site, binding energy for the miRNA-target duplex, and the evolutionary conservation of the target site sequence and position in aligned UTRs of homologous genes found in human, mouse and rat [41]. This algorithm was used to scan the human miRNA sequences against the 3′-UTRs of all the human transcripts for potential target sites. Each predicted miRNA-target duplex is assigned a binding energy (a negative value) with the cut-off value being set to −12, namely, only those sites with binding energies less than −12 were considered to be the target sites.

The predicted binding energy reflects the binding potential of the miRNA to the target site: a lower binding energy indicates a higher binding potential. Based on this data, we generate a binding score matrix *B* = (*b_ij_*)*_N_*_×*M*_, where M is 211, the number of known human miRNAs, and N is 4896, the number of genes that contain at least one target site of the 211 miRNAs. The binding score *b_ij_* is the sum of absolute values of binding energies for all the target sites of miRNA *j* within the 3′-UTR of gene *i*. If gene *i* contains no target site of miRNA *j*, we set *b_ij_* to zero. Some human genes may correspond to multiple mRNA transcripts and in this case the binding scores of these transcripts are averaged.

### Gene expression data in miRNA transfected cells

In this paper, three independent miRNA transfection data sets are used, which are originally generated by Lim et al. [23], Wang et al. [24], and Linsley et al. [25], respectively. To investigate the influence of miRNAs on gene expressions, Lim et al. transfected exogenous miRNAs into the HeLa cells and measured gene expression changes of genes in the transfected cells versus non-transfected cells using two-channel Agilent microarrays [23]. This data set is available from the GEO database with the accession number GSE2075 [40].

In total, the effect of six miRNAs were examined in the data, including two wild-type miRNAs (miR-1 and miR-124), two mutant miRNAs of miR-124 (124mut5-6 and 124mut9-10), and two chimeric miRNAs (chimiR-124/1 and chimiR-1/124). The two mutant sequences of miR-124 were designed by switching bases at positions 5 and 6 (124mut5-6) and at positions 9 and 10 (124mut9-10), respectively. ChimiR-124/1 and chimiR-1/124 were two hybrid miRNAs. ChimiR-124/1 consists of the 5′-segment (10 bases) of miR-124 and the 3′-segment (12 bases) of miR-1, while chimiR-1/124 consisted of the 5′-segment (10 bases) of miR-1 and the 3′-segment (11 bases) of miR-124. These six miRNAs were transfected separately into HeLa cells and expression changes for more than 20,000 transcripts were measured at the 12 (12 h) and 24 (24 h) hours after transfection, resulting in 12 expression change profiles. In this data set some transcripts correspond to the same gene, namely, they have the same gene symbol. By averaging the measurements of transcripts corresponding to common genes, we finally obtained the relative expression levels of 15,223 different genes for each expression profile.

In the second data set, gene expressions were profiled in miR-124 transfected as well as non-transfected HepG2 cells at 7 time points using the Affymetrix GeneChips [24]. We compared the expression profiles in transfected cells with those in non-transfected cells at each time point to obtain 7 expression change profiles. That is, these expression change profiles measure the relative expression levels (log ratios) of genes in miR-124 over-expressed cells with respect to non-transfected controls. The third data set contains four expression change profiles, which measure the relative expression levels of genes in miR-16 and miR-106b over-expressed and inhibited Hela cells using the two channel Agilent microarrays (non-transfected cells as controls) [25]. For both of these two data sets, multiple measurements for the same transcripts were averaged to represent the relative expression levels of genes.

### Inferring relative activities of miRNAs from gene expression data

To calculate the relative activities of miRNAs, we examine the expression changes of all genes between two different conditions measured by microarray experiments, which is denoted as “expression change profile” in this article. The expression changes of genes are directly measured by cDNA arrays or calculated by comparing absolute gene expression levels captured by pairs of oligonucleotide arrays. Given an expression change profile *e* = (*e_1_*,*e_2_*, …, *e_N_*) (e.g. expression changes of genes in mutant versus wild-type cells, in which *e_i_* is the log ratio of gene i) and a binding score matrix *B* = (*b_ij_*)*_N_*_×*M*_ (e.g. the above described binding score matrix for known human miRNAs), where *N* and *M* are the total number of genes and miRNAs respectively, our method aims to identify the miRNAs that are associated with the gene expression changes in *e*. The basic idea is to examine the expressions of target genes of miRNA: if the target genes tend to be down-regulated, we infer its activity to be enhanced; conversely, if the target genes tend to be up-regulated, we infer its activity to be repressed.

For each miRNA, we calculate a score, termed the activity change score (AC score), which reflects the activity change of the miRNA. Suppose that we extract the binding scores corresponding to the current considered miRNA from the matrix B, and denote it as *b* = (*b*_1_, *b*_2_, …, *b_N_*). To calculate the AC score, we first sort the expression vector *e* in a decreasing order and denote the sorted expression vector as *e*′ = (*e*_(1)_, *e*_(2)_, …, *e*_(*N*)_). Accordingly, we rearrange the binding vector *b* into 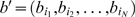, where (*i*_1_, *i*_2_, …, *i_N_*) are the indices of the ranked genes in *e*′. Note that 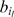 and *e*_(*l*)_ correspond to the same gene *i_l_*.

Second, we combine the two vectors *e*′ and *b*′ into a non-decreasing function *f*(*i*) defined as follows:
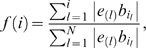
(1)where 1≤*i*≤*N*. Meanwhile we define another increasing function *g*(*i*) based only on *e*′ as
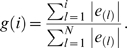
(2)If the activity of a miRNA does not change, genes with high and low binding affinities to the miRNA would locate randomly in the ranking list *e*′, and *f*(*i*) would increase in a stochastic manner. However, if the activity of a miRNA does change, for example in a down-regulated manner, genes with high binding affinities would rank high in *e*′, and *f*(*i*) would increase rapidly. The function *g*(*i*) serves as a control for comparison with *f*(*i*).

Third, we search for the index *i_max_* that achieves the maximum deviation between *f*(*i*) and *g*(*i*), that is, 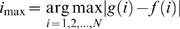. Then a pre-score is defined as
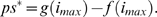
(3)Fourth, the pre-score is treated as a statistic and permutations are performed to obtain its distribution under the null hypothesis: no association between *e* and *b*. The binding vector *b*′ is permutated *K* times, resulting in *K* permuted binding vectors *b* ^(1)^, *b* ^(2)^, …, *b* ^(*K*)^. For each of the permutated binding vectors, we calculate a permutated pre-score and thereby we obtain a permutated pre-score vector denoted as *ps^perm^* = (*ps* ^1^, *ps* ^2^, …, *ps^K^*). The vector *ps^perm^* represents the distribution of the pre-score under the null hypothesis and will be used for two purposes: (1) normalizing the pre-score and (2) assessing the significance of the activity change of a miRNA (to be described in the next section).

**Figure 6:**
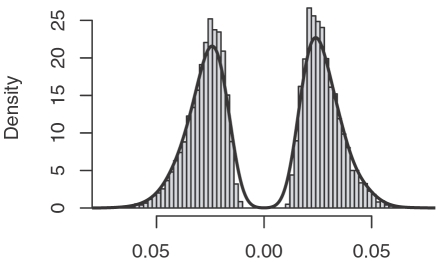
Distribution of the pre-scores for miR-124 in 10,000 permutated data sets.

Since the pre-scores of different miRNAs may have different distributions under the null hypothesis, they are not directly comparable. Therefore, we normalize the pre-score into the AC score defined as the following:
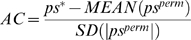
(4)where *MEAN*(*ps^perm^*) is the mean of *ps^perm^* and *SD*(|*ps^perm^*|) is the standard deviation of the absolute values of *ps^perm^*. In General, the permutated pre-score has a bimodal distribution as shown in Figure 6. It can be shown that if the expression change profile *e* is symmetric with respect to zero, the permutated pre-score would also have a symmetric distribution. In most microarray data, the symmetric assumption for the expression change profile *e* is approximately satisfied and therefore we use *SD*(|*ps^perm^*|) to combine the standard deviations of the permutated pre-scores in the positive and negative sides. Certainly, the symmetry is not perfect: the distribution of the pre-scores may skew to one side. We use *MEAN*(*ps^perm^*) to correct the skewness, which is usually close to zero. In practice, the AC score achieves a good normalization for the pre-scores of different miRNAs. The AC score can be either positive or negative: a positive value indicates an overall down-regulation of the target genes of a miRNA and thereby the enhanced activity of the miRNA. Conversely, a negative value indicates an overall up-regulation of the target genes of a miRNA and thereby the reduced activity of the miRNA.

The AC score is a generalization of the enrichment score in gene set enrichment analysis (GSEA), which was proposed to identify gene sets associated with expression change profiles [35]. A target gene set of a miRNA, can be regarded as a degenerated binding score profile, in which binding scores above and below a specified threshold are set to 1 and 0, respectively. In this situation, our method is similar to the GSEA method. More explanations and applications of the method in the context of transcription inference can be found in [44], [49].

### Significance evaluation of AC scores

The significances for the AC scores can also be assessed based on the above described permutation results. We define the p-value as the percentage of permutations that result in equal or more extreme pre-scores than the one from the original data. That is, the p-value for *ps* ^*^ is defined as
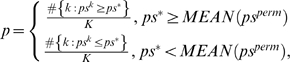
(5)where *MEAN*(*ps^perm^*) is the mean of *ps^perm^* and *K* is the number of permutations.

Since usually hundreds of miRNAs are examined simultaneously for each expression profile, multiple testing corrections need to be considered. Therefore, we calculate the false discovery rate (FDR) based on the permutations using a similar method introduced in GSEA [35]. The AC scores for each miRNA are calculated in both the original data, denoted as *ACS*(*r*) for the *r_th_* miRNA, and the permutated data, denoted as *ACS*(*r*, *k*), for the *r_th_* miRNA in the *k_th_* permutation. We then consider the histogram of all *ACS*(*r*, *k*) over all *r* and *k*, and use this null distribution to compute an FDR *q* value for a given AC score *ACS*(*r*) = *ACS* ^*^. If *ACS* ^*^≥0, the FDR is the ratio of the percentage of all (*r*, *k*) with *ACS*(*r*, *k*)≥0, whose *ACS*(*r*, *k*)≥*ACS* ^*^, divided by the percentage of miRNAs with *ACS*(*r*)≥0, where *ACS*(*r*)≥*ACS* ^*^, and similarly if *ACS* ^*^<0.

### Software availability

The C++ program for the method is available for downloading at http://leili-lab.cmb.usc.edu/yeastaging/projects/microrna/.

## Supporting Information

Table S1 AC scores, p-values and q-values for 211 human miRNAs in expression profiles from miRNA transcfection experiments performed by Lim et al. [23].
(0.16 MB XLS)

Table S2 AC scores, p-values and q-values for 211 human miRNAs in expression profiles from miRNA transcfection time course experiments performed by Wang et al. [24].
(0.10 MB XLS)

Table S3 AC scores, p-values and q-values for 211 human miRNAs in expression profiles from miRNA transcfection and inhibition experiments performed by Linsley et al. [25].
(0.13 MB XLS)
